# Balance impairment in myotonic dystrophy type 1: Dynamic posturography suggests the coexistence of a proprioceptive and vestibular deficit

**DOI:** 10.3389/fnhum.2022.925299

**Published:** 2022-07-28

**Authors:** Stefano Scarano, Valeria Ada Sansone, Carola Rita Ferrari Aggradi, Elena Carraro, Luigi Tesio, Maurizio Amadei, Viviana Rota, Alice Zanolini, Antonio Caronni

**Affiliations:** ^1^Department of Biomedical Sciences for Health, University of Milan, Milan, Italy; ^2^Department of Neurorehabilitation Sciences, IRCCS Istituto Auxologico Italiano, Ospedale San Luca, Milan, Italy; ^3^NEuroMuscular Omnicentre, Fondazione Serena Onlus, Milan, Italy

**Keywords:** myotonic dystrophy, balance, posturography, falls, neurological rehabilitation

## Abstract

Falls are frequent in Myotonic Dystrophy type 1 (DM1), but the pathophysiology of the balance impairment needs further exploration in this disease. The current work aims to provide a richer understanding of DM1 imbalance. Standing balance in 16 patients and 40 controls was tested in two posturographic tests (EquiTest™). In the Sensory Organization Test (SOT), standstill balance was challenged by combining visual (eyes open vs. closed) and environmental conditions (fixed vs. sway-tuned platform and/or visual surround). In the “react” test, reflexes induced by sudden shifts in the support base were studied. Oscillations of the body centre of mass (COM) were measured. In the SOT, COM sway was larger in patients than controls in any condition, including firm support with eyes open (quiet standing). On sway-tuned support, COM oscillations when standing with closed eyes were larger in patients than controls even after taking into account the oscillations with eyes open. In the “react” paradigm, balance reflexes were delayed in patients. Results in both experimental paradigms (i.e., SOT and react test) are consistent with leg muscle weakness. This, however, is not a sufficient explanation. The SOT test highlighted that patients rely on vision more than controls to maintain static balance. Consistently enough, evidence is provided that an impairment of proprioceptive and vestibular systems contributes to falls in DM1. Rehabilitation programs targeted at reweighting sensory systems may be designed to improve safe mobility in DM1.

## Introduction

The clinical hallmarks of the Myotonic Dystrophy type 1 (i.e., DM1) are muscular weakness and wasting, myotonia and early-onset cataracts (Harley et al., 1993). Weakness typically develops in the face, neck, and distal muscles of the upper and lower limbs. DM1 is a generalized disorder rather than an isolated affection of muscles. Gastrointestinal symptoms and endocrine disorders are typically present, and diffuse changes in the white matter can be found at the brain level. Dysexecutive features and apathy are also characteristic (Ashizawa, 1998; Serra et al., 2014).

Gait impairment, common in DM1, has been attributed to several factors. Patients have reduced walking speed or some gait irregularities (Bachasson et al., 2016). Stumbles and falls for unknown reasons are very frequent in this disease (Wiles et al., 2006). It has been estimated that the fall risk of DM1 patients shows a 10-fold increase compared to age-matched controls (Wiles et al., 2006). In addition, up to 20% of falls in DM1 result in fractures and, as in other pathological conditions, falls cause anxiety about falling and disability in DM1 patients.

The origin of the balance impairment in DM1 has not been fully clarified yet (Hammarén et al., 2014).

In the first place, muscular weakness plays a crucial role in causing balance impairment and increasing the risk of falling in DM1. Muscular weakness appears to influence the total number of falls, the likelihood of recurrent falls, and dangerous falls (Horlings et al., 2008; Hammarén and Kollén, 2021). A pathophysiological theory has been put forward according to which weakness of the distal lower limb muscles (e.g., ankle extensors) increases the risk of stumbling. In contrast, proximal weakness (e.g., hip flexors) increases the risk of falling (Horlings et al., 2008).

However, regarding DM1, it seems unlikely that the balance impairment is attributable to weakness only. For example, although myotonia in the lower limbs is not a typical complaint in patients with DM1, it can be found during the EMG examination (Logigian et al., 2007), and we cannot rule out it may also play a role by increasing muscle stiffness.

In addition, it is well-known that not only muscular force but also inputs from proprioceptors, eyes, and vestibulum are needed for balance (Fitzpatrick and McCloskey, 1994). As evidence of this, it is enough to mention that patients with impaired proprioception from the lower limbs, like those with peripheral neuropathy, suffer an increased fall risk (Richardson and Hurvitz, 1995). Likewise, fall risk increases in patients with a vestibular deficit (Herdman et al., 2000). Finally, poor sight is also a risk factor for falls, for example, in the elderly (Harwood, 2001). In this respect, various somatosensory deficits have been suggested in DM1, which could also contribute to balance impairment (Bachasson et al., 2016). For example, in more than 10% of DM1 patients, an actual peripheral neuropathy has been demonstrated (Hermans et al., 2011), and a conduction disturbance along the dorsal column–medial lemniscus pathway (i.e., the spinal somatosensory tract) has also been found in myotonic dystrophies (Gott and Karnaze, 1985).

The need for a richer understanding of the balance impairment in DM1 has been highlighted (Hammarén et al., 2015). In this regard, dynamic posturography, which consists of recording the body sway in perturbed balance conditions through force platforms, provides a valuable insight into the pathophysiology of balance disorders (Bloem et al., 2003). More precisely, in dynamic posturography, standing subjects are perturbed (for example, by a motion of the supporting platforms) so that keeping the upright stance becomes more challenging. By administering appropriate balance disturbances, it is possible to stress the different systems involved in balance regulation (e.g., proprioceptive and vestibular systems, postural reflexes) to infer their functioning in the tested individual.

To our knowledge, only one study (Missaoui et al., 2010) assessed DM1 patients with posturography. However, this previous work was more aimed at evaluating the effects of rehabilitation on balance rather than at describing the characteristics of the balance impairment of these patients.

Based on the above, the current work aims to detail the pathophysiology of the balance impairment in DM1. In particular, because of the systemic involvement of the disease and since sensory deficits have been suggested in DM1, we hypothesized the impairment of the proprioceptive and vestibular systems involved in standing balance regulation. Hence the role of proprioceptive, visual, and vestibular contributions to balance in a cohort of well-characterized adult patients with DM1 was investigated. To this aim, dynamic posturography was used.

## Materials and methods

This is an observational, cross-sectional study. From October 2020 to September 2021, 16 DM1 patients and 40 healthy controls were consecutively recruited. The current work is part of an ongoing study investigating an association between poor balance and cervical proprioception in DM1 (ClinicalTrials.gov: NCT04712422): the study complied with the Declaration of Helsinki and was approved by the ethical committee of the IRCCS Istituto Auxologico Italiano. All participants gave their informed consent to participate in the research.

### Participants

Patients were recruited according to the following criteria.

#### Inclusion criteria

1. Genetically confirmed patients with DM1 [E1 (CTG repeats: 50–150)], E2 (150–1000), and E3 (>1000) (New nomenclature and DNA testing guidelines for myotonic dystrophy type 1 (DM1). The International Myotonic Dystrophy Consortium [IDMC], 2000);
2. Age > 18;
3. Ability to keep the upright stance without assistance and assistive devices for at least 20 s;
4. Rivermead Mobility Index (Antonucci et al., 2002) score ≥ 10/15. In the absence of validated cut-off levels for DM1, this level was adopted because it represents two standard measurement errors above the average discharge scores of stroke patients after inpatient rehabilitation.
5. Visual acuity > 10/20, corrective lenses allowed;
6. Mini-Mental State Examination (Folstein et al., 1975) score ≥ 26/30.

#### Exclusion criteria

1. Any balance impairment caused by a neurological or cardiovascular disease, or muscular-skeletal disorder, or other pathological conditions which, according to the principal investigator, could affect the results of the tests to be performed;
2. Pregnancy;
3. Any previous orthopedic surgical intervention;
4. Head or neck trauma in the 6 months preceding the study.

The healthy controls were included if aged > 18 and shared the patients’ same exclusion.

Outpatients were recruited from the NeuroMuscular Omniservice Clinical Center (NEMO) in Milan, Italy, a dedicated Clinical Center for patients with neuromuscular diseases and experience in Myotonic Dystrophies. Controls were recruited among the personnel and the visitors of the Department of Neurorehabilitation Sciences, IRCCS Istituto Auxologico Italiano in Milan.

Patients were seen at the NEMO Center by two independent neurologists (VS and AZ) or a physiatrist (EC) and enrolled with no a priori selection if they complied with the inclusion and exclusion criteria described above. They came into the clinic for routine outpatient assessments. No patient had physical or occupational therapy sessions within 3 months before the study enrollment. In addition, no patient ever participated in rehabilitation trials to improve their gait or balance disorder. Patients were encouraged by their treating physicians to stay physically active (e.g., through outdoor walking).

Patients received a full clinical assessment and an instrumental balance assessment on the EquiTest posturographic instrument. Controls only received posturography.

### Clinical assessment

Clinical and demographic data were recorded at baseline (Table 1). In addition to a general clinical examination, patients were assessed for the severity of their muscle impairment, mobility, and perception of dizziness.

**TABLE 1:** Patients’ characteristics.

ID	Age, years	Gender	Height, m	Duration, years	E class	MIRS	DHIsf	RMI	n of falls
1	41	F	1.70	15	2	3	13	15	0
2	39	F	1.68	26	1	3	11	15	2
3	25	F	1.62	11	2	3	13	15	3
4	42	M	1.78	11	1	3	10	15	0
5	40	M	1.82	21	1	1	13	15	0
6	26	M	1.69	25	2	3	13	15	0
7	21	F	1.70	19	3	2	12	15	3
8	47	M	1.65	42	1	4	9	15	1
9	43	F	1.56	28	2	3	11	10	5
10	42	M	1.78	23	2	3	10	12	0
11	47	F	1.65	14	2	4	13	14	0
12	47	F	1.60	21	2	4	13	14	0
13	46	F	1.60	17	2	3	8	12	1
14	41	F	1.64	31	3	3	9	14	1
15	38	F	1.70	26	2	4	13	14	2
16	47	M	1.74	19	2	3	11	14	0
PTS	41.5 (21–47)	F/M: 10/6	1.69 (1.56–1.82)	21 (11–42)	2 (1–3)	3 (1–4)	11.5 (8–13)	14.5 (10–15)	0.5 (0–5)
CNT	35 (26–57)	F/M: 22/18	1.69 (1.50–1.90)	−	−	−	−	−	0 (0–0)

Clinical and anthropometric characteristics of each of the 16 patients recruited in the study. The last two rows report the median values (and range, in brackets) of the patients’ (PTS) and controls’ (CNT) sample. The ratio between the number of females and the number of males (F/M) is also given. ID, patients’ identification number; F, females; M, males; duration, disease duration; E class, class attributed according to the number of repetitions (i.e., expansion) of CTG triplets; MIRS, Muscular Impairment Rating Scale; DHIsf, Dizziness Handicap Inventory—short form; RMI, Rivermead Mobility Index; n of falls: number of falls in the 12 months before the assessment.

The Muscular Impairment Rating Scale (MIRS) (Mathieu et al., 2001) was designed to rate the severity of the muscular impairment in DM1 and consists of a single item scored on five categories (0: no muscular impairment; 5: severe weakness of proximal muscles, e.g., hip muscles). Of note here, score 3 indicates distal weakness (e.g., leg muscles) and score 4 mild to moderate proximal weakness.

The Dizziness Handicap Inventory—short form (DHIsf) (Tesio et al., 1999) is a self-administered questionnaire (13 dichotomous items) returning an ordinal score of self-perceived unsteadiness. The total questionnaire score may range from 0 to 13 (the higher, the better). Two representative items are “Do quick movements of your head increase your problem?” and “Because of your problem, is it difficult for you to go for a walk by yourself?”

The Rivermead Mobility Index (RMI) (Antonucci et al., 2002) consists of 15 dichotomous items, evaluating different gross motor skills. Each item can be scored 1 or 0, depending on the patient’s capacity to perform an activity independently or not. The higher the RMI total score, the higher the motor competence. For example, two representative items are “Sitting to standing” (Does the patient stand up from any chair in less than 15 s, and stand there for 15 s, using hands and with aid if necessary?) and “Stairs” (Does the patient manage a flight of stairs without help?).

A fall was defined as “an unintentional or unexpected loss of balance resulting in coming to rest on the floor, the ground, or an object below knee level” (Logan et al., 2021). The total number of falls in the 12 months before the assessment was also recorded.

### Instrumental assessment: Posturography

Computerized posturography (EquiTest^®^, Neurocom International Inc., Clackamas, OR, United States) was used for balance assessment.

The EquiTest^®^ instrument consists of two integral force platforms connected to electric engines. The platforms are nested within a large, curved screen surrounding the front and lateral sides of the subject standing on the platforms. Steered by dedicated software, in the different testing conditions, the force platforms can rotate on the sagittal plane (i.e., around the medial-lateral pitch axis) or shift horizontally along the anterior-posterior axis. The surround can only rotate on the sagittal plane (medial-lateral pitch axis) (Tesio et al., 2013).

In the current study, the Equitest^®^ instrument administered two balance tests to healthy controls and DM1 patients: the sensory organization test (SOT) and the motor control test (MCT). In both trials, the task was standing as still as possible.

#### The sensory organization test

In the SOT, the participants stand barefoot, with one foot on each platform, the ankles in line with the tilting axis shared by the platforms and the visual surround, while holding the upper limbs along the trunk. Feet are placed parallel, about 15 cm apart distance adjusted to patient’s height. A suspension harness provides safety against falls.

The SOT consists of eighteen 20-s standing trials. Six different sensory-motor conditions are imposed based on two sight modalities (eyes open or closed) and two motion modalities of the platforms and the surround [steady or moveable; Figure 1 from Scarano et al. (2022)].

**FIGURE 1:**
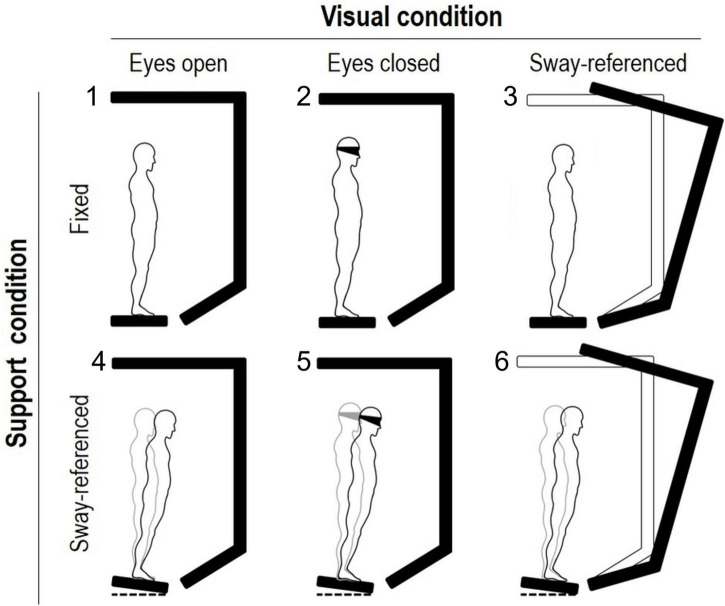
Experimental setup of the sensory organization test (EquiTest system). Two support and three visual conditions (figure’s rows and columns, respectively) are combined to obtain the six balance tasks of the sensory organization test. In the sway-referenced condition, the platform and/or the screen move in the sagittal plane after the whole-body center of mass oscillation. Keeping balance is more and more challenging from conditions 1 (eyes open, fixed) to 6 (eyes open with sway-referenced screen and sway-referenced platform). Three 20-s trials are completed in each of the six tasks.

As customary in balance studies (Nashner et al., 1982; Nashner and Peters, 1990), Equitest^®^ posturography considers the standing subject as an inverted pendulum hinged at the ankle. Based on this, a dedicated algorithm estimates the angular oscillations of the body centre of mass (COM) in the sagittal plane from the ground reaction forces and the subject’s height.

The subject’s performance during each SOT trial is indicated by the equilibrium score, which is calculated as follows:


(1) $$equilibriumscore=100\times \left(1-\frac{\theta_{ant}-\theta_{post}}{12.5}\right)$$

with θ_*ant*_ and θ_*post*_ indicating the maximum anterior and posterior COM displacement during the trial, respectively. θ is the angle between a vertical line projecting upward from the center of the base of support and a line connecting this point to the COM (Bonan et al., 2004). The 12.5° comes from the sum of the limits of stability of the COM, i.e., the maximum anterior and posterior angles (8.5° and 4°, respectively) that the COM can sway on the sagittal plane without losing balance (Nashner and Peters, 1990). The equilibrium score ranges from 0 to 100, with 100 indicating no COM sway (unattainable) and 0 flagging an “Equitest fall.” Such a “fall” implies oscillations beyond the limits of stability, compensatory steps, or arms touching the surrounding (“parachute reactions”) during the trial.

The mechanism moving the platforms or the surround in the SOT is peculiar since sagittal tilts of the platforms or the visual surround are driven by COM oscillations. This “sway-referenced” motion of the platforms is calibrated so that the ankles remain in their neutral position despite the COM sway. For example, a forward displacement of the COM triggers the downward tilt of the platforms and (almost) no ankles’ rotation occurs despite the COM motion. Eventually, leg *proprioceptors falsely signal* body stability (Peterka and Benolken, 1995). In the same vein, the surround’s sway keeps the distance from the surround to the eyes stable. Thus, *vision falsely signals* body stability.

Each 20 s trial of the six SOT conditions is repeated three times. In SOT conditions 1 and 2, eyes are open and closed. In condition 3, the eyes are open, but the surround is sway-tuned. Finally, conditions 3, 4, and 5 replicate conditions 1–3, but the platform is also sway-tuned. Hence, in conditions 1, 2, and 3, subjects standstill on the fixed platforms, while in tasks 4, 5, and 6, the platforms oscillate.

Equilibrium scores are averaged in each sensory-motor condition across the three trials.

#### The motor control test

In the MCT, the upright stance is perturbed by a sudden shift in the transverse plane of both platforms forward and backwards. Three stimuli intensities are tested (small, medium, and large), each repeated three times.

During this test, participants stand on the force platforms with their feet about 15 cm apart (distance adjusted to the patient’s height), eyes open, and arms along the body. The subject is requested to stand still with eyes open and balance without stepping or touching the surround.

In this test, the stimulus intensity is strong enough to evoke the reflex contraction of the leg muscles (Perucca et al., 2014). For example, a backward shift causes the ankles’ dorsiflexion and thus the brisk elongation of plantar flexors muscles, which triggers their contraction and eventually changes the ground reaction forces. The latency of this change in the reaction forces (i.e., the latency of the balance reflexes as seen by the platforms; reflex torques) is finally measured for each limb.

The amplitude of the platform shift was scaled with the participant’s height so that small, medium, and large perturbations caused 0.7°, 1.8°, and 3.2° COM displacements in the sagittal plane with respect to the base of support, respectively (Vanicek et al., 2009). For a 1.8 m tall individual, these angular displacements correspond to platform translations of about 1.5, 3, and 6 cm (Tesio et al., 2013). The duration of the platform shift also increased in the three MCT conditions (small perturbation: 250 ms; medium: 300 ms; large: 400 ms), as well as the angular velocity of the COM displacement (Vanicek et al., 2009). Differently from the stimulus amplitude, the stimulus duration was independent of the participant’s height. The single platform translations occurred at a constant velocity (i.e., ramp stimulation).

After a forward or backward displacement, the platform slowly returns to its starting position. After the starting position is recovered, 1.5–2.5 s passes before a new platform shift is administered to the participant to avoid anticipation.

### Data analysis and statistics

The Equitest software automatically returns the mean equilibrium score of each of the six SOT conditions (see above) and the mean latency of the reflexes evoked in the MCT. According to the manufacturer, the automated measurement of the reflexes latency is satisfactory (International, 2008). In addition, independent scholars consider the Equitest MCT to provide reliable measures (Harro and Garascia, 2019; Carvalho et al., 2021), and our research group confirms this finding (Tesio et al., 2013).

Those blocks of three trials in which the participant fell in all three repetitions, and thus with a mean score equal to zero, were referred to as “fall blocks.” For the current work, since no difference was found between the opposite limbs in the MCT (Perucca et al., 2014), the latencies of the mechanical reflexes recorded by the two force platforms were averaged.

Demographic and clinical characteristics were summarized with median and range. The Wilcoxon rank-sum test compared age and height in patients and controls. Fisher’s exact test for count data was used to compare gender distribution in the two groups.

Linear mixed-effects models were used to test differences between groups and the various experimental conditions of the SOT and the MCT. The SOT and the MCT are complete factorial experiments. In the SOT, the effects on the different equilibrium scores (dependent variable) of the support condition (fixed vs. sway-referenced platforms), sight (eyes open vs. eyes closed vs. sway-referenced screen) and their interaction were tested. In the analysis of the MCT, the dependent variable was the latency of the reflex torques. The direction of the platforms’ shift (forward vs. backward), the perturbation amplitude (small vs. medium vs. large) and their interaction were evaluated. In addition to these *within-group* differences, *between-group* differences (i.e., controls vs. DM1 patients) and the interaction across within- and between-group factors were assessed. As recommended, maximal models were tested, i.e., models including full random slopes and intercepts (Barr et al., 2013).

The linear models’ assumptions of normally distributed and homogeneous residuals were checked graphically with the quantile-quantile plot and residual/predicted plot, respectively (Faraway, 2016). Since these assumptions were violated in all the models tested here, the response variables were transformed. The 0–100 equilibrium score of the SOT was log-transformed into *ln*⁡(100−*ES*), with (100−*ES*) indicating the amplitude of the COM sway. Thus, contrary to the original formulation, higher values of the converted equilibrium score indicate poorer balance.

The reciprocal transformation was needed for reflexes’ latencies to obtain normality and homoscedasticity. The transformed variable, called “immediacy,” should be interpreted similarly to a reflex conduction velocity (the higher its numerical value, the better).

Type III analysis of variance with Satterthwaite’s method evaluated fixed effects’ significance and interactions. The least-squares means were calculated for *post hoc* testing and graphical purposes. Satterthwaite’s approach has also been used for *post hoc* testing (Luke, 2017).

The significance level was set at 0.05. Finally, the Bonferroni correction was applied to the *post hoc* tests.

Statistical analyses were run in R version 3.6.2.

## Results

Most patients were affected by distal weakness, with 14 (out of 16) scoring ≥ 3 on the MIRS (Table 1). Eight out of 16 patients reported at least one fall 12 months before study enrolment, and five were recurrent fallers. None of the controls had fallen in the same period. Nine patients scored ≤ 12 on the DHIsf, indicating that about half of patients complained about self-perceived unsteadiness.

No difference was found in terms of age (median, range) between patients (41.5 years, 21–47 years) and controls (35 years, 26–57 years; Wilcoxon rank-sum test: *p* = 0.141). The gender distribution (number of females vs. males) was comparable between the two groups (patients: 10 vs. 6; controls: 22 vs. 18; Fisher’s exact test: *p* = 0.767). Height was also superimposable in patients (1.69 m, 1.56–1.82 m) and controls (1.69 m, 1.50–1.90 m; Wilcoxon rank-sum test: *p* = 0.567).

### Sensory organization test

**FIGURE 2:**
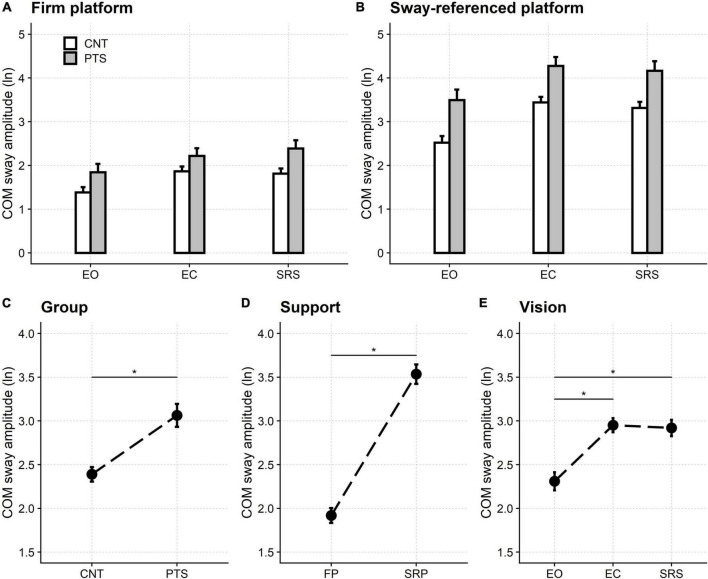
Sensory organization test. Upper row: natural logarithm (ln) of the normalized amplitude of the sway of the centre of mass (COM) when standing on the firm **(A)** and the sway-referenced platforms **(B)**. Lower row: main effects of the regression model. The ln of the normalized COM sway amplitude is shown for the two groups of participants [**(C)** Group], the two support conditions [**(D)** Support], and the three visual conditions [**(E)** Vision]. Even if significant, the model’s interactions did not alter this pattern of significance (see main text and Supplementary material). CNT, controls; PTS, patients; EO, eyes open; EC, eyes closed; SRS, sway referenced screen; FP, firm platform; SRP, sway-referenced platform. Mean and 95% confidence interval are plotted; *a significant difference.

The balance was poorer in patients and controls when standing on the unstable platform than with fixed support (Figure 2). In addition, in both groups and standing conditions, the sway increased when the subject was standing with the eyes closed or eyes open with the sway-referenced screen compared to standing with the eyes open. Finally, patients performed worse than controls in all six sensory-motor conditions.

The statistical analysis of significance supported these findings.

All three main effects were significant in the analysis of variance: participant’s group [*F*_(1,54.0)_ = 75.97, *p* < 0.001], visual condition [*F*_(2,80.6)_ = 136.51, *p* < 0.001], and support condition [*F*_(1,54.0)_ = 676.24, *p* < 0.001].

The COM sway was significantly larger in patients (3.06, 95% CI: 2.93–3.20) than controls (2.39, 95% CI: 2.31–2.47) and when standing on the unstable platforms (3.54, 95% CI: 3.42–3.65) compared to standing on firm ones (1.92, 95% CI: 1.83–2.00).

*Post hoc* testing showed that, compared with the open-eyes stance (2.31, 95% CI: 2.21–2.41), the COM sway was significantly more considerable (*p* < 0.001) when standing with the eyes closed (2.95, 95% CI: 2.87–3.03) and while looking at the sway-referenced screen (2.92, 95% CI: 2.83–3.01). No difference was found between standing with closed eyes and the sway-referenced screen (*p* = 1.000).

Interactions were significant both between the participants’ groups and the support conditions [*F*_(1,54.0)_ = 1.58, *p* = 0.001], and between sight and support [*F*_(2,162)_ = 13.35, *p* < 0.001], but this did not affect the significance pattern pointed out by the main effects, described above.

Nine patients had at least one fall block, all nine in the moving platform condition (two in the eyes open, eight in the eyes closed and nine in the sway-referenced vision conditions, respectively). No control participant fell. Results were confirmed by a control analysis in which the Equitest “fall blocks” were neglected.

This control analysis and the complete regression analysis results reported above can be found in Supplementary material.

### Motor control test

**FIGURE 3:**
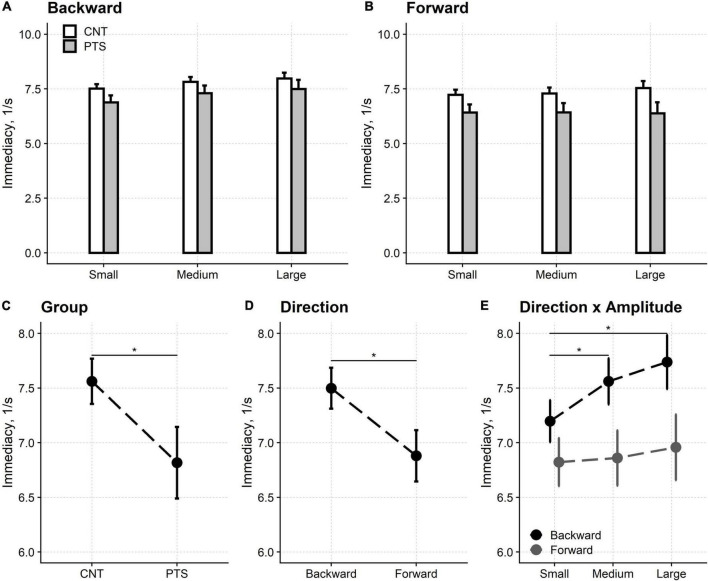
Motor control test. Upper row: the immediacy of the reflexes elicited by backward **(A)** and forward **(B)** platforms’ displacements. Lower row: main effects and interaction from the regression model. The immediacy of the force reflexes is shown for the two groups of participants [**(C)** Group] and the two directions of the platforms’ shifts [**(D)** Direction]. The amplitude of the perturbation also affected the reflexes’ immediacy. However, this is not shown because the interaction between direction and amplitude [**(E)** Direction × Amplitude] showed that the perturbation’s amplitude only affected the immediacy of the reflexes evoked by backward shifts. CNT, controls; PTS, patients. Mean and 95% confidence interval are plotted; *Significant difference at *p* < 0.05. The difference between the reflexes’ immediacy evoked by backward and forward shifts is significant for each of the three perturbations’ amplitudes (not marked with * for graphical reasons).

The immediacy of the reflex torques evoked by sudden platform shifts was reduced compared to controls for all three perturbations’ amplitudes (Figure 3). Furthermore, in both controls and patients, the reflexes’ immediacy was lower (i.e., higher latencies were recorded) in shift forward compared to shift backward conditions, respectively.

Regression analysis showed that all three main effects were significant, i.e., participant’s group [*F*_(1,54.1)_ = 14.87, *p* < 0.001], shift direction [*F*_(1,54.0)_ = 50.37, *p* < 0.001] and shift amplitude [*F*_(2,78.3)_ = 5.81, *p* = 0.004]. Immediacy was worse in patients (6.82 1/s, 95% CI: 6.49–7.14 1/s) compared to controls (7.56 1/s, 95% CI: 7.36–7.77 1/s) and in shift forward (6.88 1/s, 95% CI: 6.65–7.12 1/s) compared to shift backward (7.50 1/s, 95% CI: 7.31–7.69 1/s).

*Post hoc* tests showed that reflexes immediacy was reduced for small (7.01 1/s, 95% CI: 6.83–7.19 1/s) compared to medium (7.21 1/s, 95% CI: 7.00–7.42 1/s; *p* = 0.022) and large (7.35 1/s, 95% CI: 7.09–7.60 1/s; *p* = 0.004) perturbations. No difference was found between medium and large perturbations (*p* = 0.120). However, the interaction between direction and amplitude was significant [*F*_(2,161.5)_ = 7.47, *p* < 0.001] and *post hoc* testing highlighted that the differences in reflexes immediacy between the three perturbation’s amplitudes are only valid for backward shifts.

The interaction between the participants’ group and the direction of the platforms’ shift was significant [*F*_(1,54.0)_ = 5.25, *p* = 0.026]. Nevertheless, this finding did not affect the pattern of significant main effects above (see Supplementary material).

### Sensory control of balance in myotonic dystrophy type 1: Additional analyses of the sensory organization test

The SOT showed that the COM sway was larger in patients than controls even when standing with the eyes open, both on firm and sway-tuned platforms. Therefore, it is possible that the between-group differences found in the closed-eyes and the sway-referenced screen conditions just reflected the between-groups difference in quiet standing, whichever the added visual state.

**FIGURE 4:**
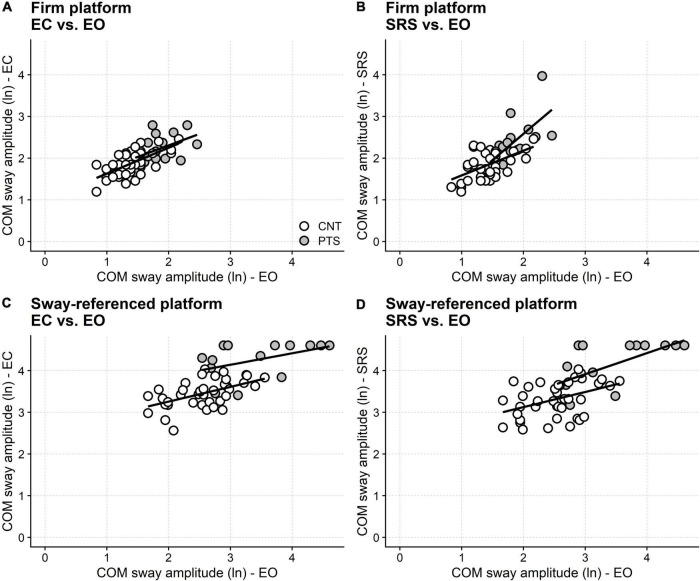
Visual dependence in DM1. Results from the SOT test. The abscissa gives the subjects sway (natural logarithm, ln, of the normalized COM oscillations) with eyes open, both in controls (open circles) and patients (filled circles). The ordinate gives the sway amplitude in different conditions (see panel labeling). Linear regression lines calculated separately for controls (CNT) and patients (PTS) are also shown. Regression predicts that standing with closed eyes **(A)** and with sway-referenced surround **(B)** does not differ between patients and controls on firm support. By contrast, given the same performance with the eyes open on the sway referenced platform, patients perform worse than controls with eyes closed **(C)** and with the sway referenced surround **(D)**. This finding points out “visual dependence” (i.e., the excessive reliance on visual information in keeping balance) and suggests difficulty using the vestibular or the proprioceptive information for regulating balance, at least in the most challenging conditions. EO, eyes open; EC, eyes closed; SRS, sway-referenced surrounding screen.

On the firm platform, once the COM sway with the eyes open is included in the regression model, no difference is found anymore between the amplitude of the patients’ sway and that of controls in both eyes closed (*F*_1_ = 0.67, *p* = 0.417) and sway-referenced screen (*F*_1_ = 3.27, *p* = 0.076) conditions (Figure 4).

In contrast, when standing on the moving platforms, the difference between patients and controls still holds in both the eyes closed and the sway-referenced screen conditions after conditioning out the sway amplitude with the eyes open (eyes closed: *F*_1_ = 17.55, *p* < 0.001; sway-referenced screen: *F*_1_ = 8.13, *p* = 0.003). Furthermore, this difference still holds for the eyes-closed condition when the fall blocks are removed from the analysis. By contrast, when fall blocks are removed, no between-groups difference is found anymore when standing with the sway-referenced screen (Supplementary material), likely because of the reduced sample size.

## Discussion

In the current study, the SOT and the MCT test paradigms of EquiTest posturography were used to evaluate static balance in DM1. The SOT showed that the amplitude of the COM sway is larger in DM1 patients than controls, even in quiet standing with the eyes open, and the MCT highlighted that balance reflex torques were slower in patients.

The most immediate interpretation of these results is that the patients’ imbalance is caused by leg weakness, which is common in DM1 (Hammarén and Kollén, 2021), and it affects most patients recruited here. The results of the MCT seem consistent with this interpretation. The MCT demonstrates that reflex torques in patients and controls after forward platforms’ displacements were delayed than those evoked by a backward shift. The reflex torques produced by the platform’s displacements are generated by the contraction of the leg muscles, with the forward and the backwards shift evoking the reflex activation of the Tibialis and Triceps surae muscles, respectively. To note here, the latency of the electromyographic reflexes evoked in the Tibialis anterior by forwards shifts is the same as that of the reflexes evoked in the Gastrocnemius medialis by backward displacements (Perucca et al., 2014). This finding points out that the delayed latency of the reflex torques elicited by a forward shift cannot be attributed to a longer or slower route of the reflex pathway within the nervous system. Instead, differences in the mechanical properties of muscles should be brought into play.

Muscles and tendon mechanics can affect the rate of force development. Different muscles [e.g., Tibialis anterior vs. Soleus (Buchthal and Schmalbruch, 1970)] consist of a different mixture of fast and slow muscle fibers, which have different times to maximal contraction. Force development also depends on the characteristics of the connective tissue of the muscle and the tendons, and compliant tendons decrease the rate of force delivery (Maffiuletti et al., 2016).

Regarding the delayed latency of the reflex torques in DM1 patients, it is also noteworthy that contraction time depends on the contraction’s strength and that force generation is delayed in the case of weak or fatigued muscles (Cudicio et al., 2021; D’Emanuele et al., 2021). Muscular and connective alterations are also profound in this disease. Histological studies showed an enhanced proportion of slow fibers, fibrosis and fatty deposition in muscle biopsies of patients with DM1 (Meola and Cardani, 2015).

In addition to delaying the reflex forces studied in the MCT, these peripheral mechanisms can also affect balance during the SOT. Balancing the (unstable) human body requires a continuous adjustment in the activity of the leg muscles (Di Giulio et al., 2009). If these muscles are weak and thus the rate of their force development is diminished, they can be slow in correcting the displacement of the COM, and its sway is eventually increased.

However, the SOT results also suggest that distal weakness might not be the only mechanism impairing balance in DM1. Statistical modeling showed that patients perform worse than controls when standing on the sway-referenced platform with closed eyes, net of any difference in eyes open sway between the two groups. In plain words, the regression analysis highlighted that if patients and controls had the same eyes open sway, eyes closed sway would still be more considerable in patients than controls (Figure 4C). That is, if patients and controls had the same eyes open sway, the amplitude of the COM sway increases more in patients than controls when they close their eyes. DM1 patients do not sway too much in eyes closed stance just because they already swing too much with their eyes open. On the contrary, the regression analysis demonstrates that balance with closed eyes is “genuinely” worse in DM1 patients than in controls. Similar reasoning applies to the sway-referenced screen condition (SOT condition 6; Figure 4D), and these findings point out that patients rely on vision to keep balance more than controls.

The first applications of the SOT were focused on detecting the effects of a vestibular impairment on balance. It has been shown that patients with a vestibular impairment are prone to perform poorly in SOT conditions 5 and 6. These findings lead to an implication in the clinic: poor performance in conditions 5 and 6 points toward a vestibular impairment (Nashner and Peters, 1990).

The intuition behind the SOT is intriguing. In conditions 4–6, thanks to the sway-referenced motion of the platforms, the proprioceptive information arising from ankle muscles (Di Giulio et al., 2009) falsely signals that no sway occurs. However, this assumption is likely too simplistic (Bloem et al., 2003). First, some ankles’ movement survives on the sway-referenced platform. The fact that this ankles’ rotation is below the threshold for evoking reflex torques (Nashner and Peters, 1990) does not rule out that it provides some proprioceptive input. Second, muscles’ contractions are needed to keep the upright stance and occur when standing in conditions 4–6. The activation of the leg muscles modulates, likely increasing (Roll and Vedel, 1982), the afferent discharge from the muscle spindles, eventually providing proprioceptive information for balance regulation.

Keeping balance in SOT conditions 4–6 represents an unusual balance task. The nervous system must quickly update motor commands after decoding this novel, unexpected balance condition. To this aim, sensory information is needed. Position sense needs to be maintained by up-weighting the information provided by skin receptors (Diener et al., 1984) and spindles of proximal muscles. Vestibular information must also be up-weighted (Lockhart and Ting, 2007).

Thus, it seems reasonable that in conditions 4–6, *proprioception and vestibula are both stressed* for maintaining balance (with the motor command providing a force of the appropriate amplitude and timing). This fact seems especially true for conditions 5 and 6 when vision is unavailable or unreliable. Under this scenario, it can be proposed that the poor performance of DM1 patients in tasks 5 and 6 could be due to an impairment along both the proprioceptive and the vestibular pathways.

Interestingly, it has been demonstrated that the somatosensory system is impaired in many patients with DM1, both peripherally and centrally (Jamal et al., 1986). In more than 10% of DM1 patients, an actual peripheral neuropathy has been demonstrated (Hermans et al., 2011) (another manifestation of the disease), and a conduction disturbance along the dorsal column–medial lemniscus pathway has also been found in DM1 (Gott and Karnaze, 1985). Studies dating to the seventies showed anatomical alterations of the muscle fibers constituting the spindle receptors (Swash and Fox, 1975) and a shortage of their sensory endings (Maynard et al., 1977). Reduced or absent tendon reflexes, common in DM1, have been attributed to muscles’ weakness and wasting and the damage of the muscle spindles (Messina et al., 1976). Even if the involvement of somatic afferents in DM1 is still a matter of debate, some Authors concluded that the involvement of the peripheral nervous system is *“assumed to be constantly present”* in DM1 (Fierro et al., 1998).

Although less known, vestibular alterations have also been described in DM1 (Balatsouras et al., 2013). Moreover, white matter alterations are common in this disorder (Minnerop et al., 2011), possibly affecting the transmission along the vestibular (and somatosensory) pathways. Late in the disease progression, neck muscles are also involved in DM1; therefore, their spindles could also malfunction. Given the strict connection between neck proprioceptors and the vestibular control system (Wilson et al., 1995), it can be hypothesized that the impairment of neck proprioception could mimic (or amplify) a vestibular impairment of balance.

We know of only one study in which posturography was used to assess balance in DM1 (Missaoui et al., 2010). Of interest here, the patients evaluated in this previous work also had significant difficulties with eyes-closed balance.

The current work suffers some limitations.

The patients’ sample size is small, consisting of 16 persons only. However, the experimental complexity and the fact that patients were placed in situations mimicking their instability should be considered.

The current work is strictly focused on studying static balance. Moreover, only the COM sway in the sagittal plane (i.e., the anterior-posterior direction) was considered here. However, future investigations could also consider the COM sway in the transverse plane since increased sway in the anterior-posterior and medio-lateral directions is typical when the balance is impaired (Caronni et al., 2019). In addition, another complimentary research should investigate dynamic balance, i.e., the ability to move in an upright stance and walk without falling (Caronni et al., 2018).

The main findings of the current work come from measures of COM displacements. Several other measures of static balance are available, such as those from the center of pressure or trunk acceleration (Caronni et al., 2019). However, in strict physiological terms, the measurement of COM displacements has the highest validity in balance assessment. The very nature of fall *is* the COM displacement beyond the stability limits (Bloem et al., 2003).

At the time of the study enrollment, the patients recruited here did not participate in rehabilitation programs, including physical or occupational therapy. Therefore, in line with the study’s primary aim, the current work can be considered an investigation of the pathophysiology of the balance impairment in DM1 with no interference from therapeutic exercise. However, it should also be pointed out that the exact patients’ activity level was not investigated. Hence, it cannot be excluded that the balance impairment is mitigated in the most active patients or that these patients have a particular phenotype of balance impairment. Moreover, we cannot exclude the patients’ activity levels caused a selection bias (i.e., only the most active patients agreed to participate in the study).

Despite the study’s limitations, the results provide some therapeutic hints that may help the care of these patients. The present results suggest that these patients might be treated similarly to patients with sensory ataxia (Tesio, 2010), a conclusion promoted by the finding that DM1 patients strongly rely on vision for compensating their balance impairment.

“Visual dependence” [i.e., the excessive reliance on visual information in keeping balance (Maire et al., 2017)] represents a common but sub-optimal solution to a balance impairment. First, it must be stressed that visual control is less efficient than proprioception in maintaining standing balance (Fitzpatrick and McCloskey, 1994). Second, even when some proprioceptive and vestibular input survives, visual dependence inhibits these afferents, and proper recovery is eventually hampered. In general terms, it must be remembered that the suppression of a partially impaired function is a general mechanism spontaneously put forward after a disease (Uswatte and Taub, 2005).

In the case of sensory ataxia, a treatment aim (particularly rehabilitation) is to up-weight the inhibited proprioceptive and vestibular input. Several treatments are under evaluation in DM1, and therapeutic exercise is one of them (Turner and Hilton-Jones, 2014). The current results suggest that DM1 patients would benefit from balance training with abolished vision (i.e., with the eyes closed or blindfolded) or with reduced visual cues (e.g., dim light). Vision can also be made inaccurate by donning glasses with smeared lenses, prismatic lenses or Frenzel glasses [see chapter 11 in Shumway-Cook and Woollacott (2012) for typical exercises for balance sensory retraining]. In addition, true “weaning from vision” exercises have been proposed for treating visual dependence (Hurtado et al., 2020). Finally, novel technologies could also be of help in this regard. For example, immersive virtual reality (García-Muñoz et al., 2022) could provide a unique way to manipulate visual cues.

With these exercises, patients learn to use available inputs from proprioceptors and vestibula to regulate motor command. In this framework, patients would benefit from this recovered ability in real-life situations (e.g., walking in low light), eventually decreasing their risk of falling.

## Conclusion

Myotonic dystrophy type 1 patients rely more than healthy controls on vision to keep balance during standing. It is proposed here that the balance disorder in DM1, in addition to muscle weakness, could be due to the impairment of lower limb proprioception and the vestibular system. Therefore, it seems reasonable to propose exercises that contrast visual dependence in balance regulation, to improve standing balance and reduce falls.

## Data availability statement

The raw data supporting the conclusions of this article will be made available by the authors, without undue reservation.

## Ethics statement

The studies involving human participants were reviewed and approved by IRCCS Istituto Auxologico Italiano. The patients/participants provided their written informed consent to participate in this study.

## Author contributions

SS, VS, LT, and VR contributed to the study’s design. SS, VR, CF, EC, MA, and AZ collected the data. VR processed the data and prepared the dataset. AC analyzed the dataset, prepared the figures, and wrote the first draft of the manuscript. AC, LT, SS, and VS revised the draft. AC arranged the final version of the manuscript, which was read, commented, and approved by all Authors.
