# Supplementary material for: Balance impairment in myotonic dystrophy type 1: Dynamic posturography suggests the coexistence of a proprioceptive and vestibular deficit
Source: Front Hum Neurosci. 2022 Jul 28;16:925299. doi: 10.3389/fnhum.2022.925299 (PMC9367988; doi:10.3389/fnhum.2022.925299)
Supplement: Supplementary file 1 [file Data_Sheet_1.docx]

Supplementary Materials 1

The following pages report the complete statistical analyses of the Sensory Organization Test and the Motor Control Test described in the main text and some control analyses. In all tables, numbers have been truncated to the third decimal. The star (*) marks a significant effect, interaction or contrast.

All analyses were run in R (version 3.6.2, 2019).

**List of abbreviations**

ampl: amplitude;

back: backward;

COM: centre of mass;

CNT: controls;

DenDF: denominator degrees of freedom;

df: degrees of freedom;

dir: direction;

EC: eyes closed;

EO: eyes open;

forw: forward;

Mean Sq: mean square;

NumDF: numerator degrees of freedom;

PTS: patients;

SE: standard error;

SOT: sensory organization test;

SRP: sway-referenced platform;

SRS: sway-referenced screen;

Sum Sq: sum of squares;

# Sensory Organisation Test: full dataset.

**Type III Analysis of Variance Table with Satterthwaite's method**

**Sum Sq | Mean Sq | NumDF | DenDF | F value | P-value**

group 5.859 | 5.859 | 1 | 54.020 | 75.966 | < 0.001 *

vision 21.059 | 10.530 | 2 | 80.553 | 136.512 | < 0.001 *

support 52.160 | 52.160 | 1 | 54.012 | 676.240 | < 0.001 *

group:vision 0.194 | 0.097 | 2 | 80.553 | 1.258 | 0.289

group:support 0.893 | 0.893 | 1 | 54.012 | 11.583 | 0.001 *

vision:support 2.060 | 1.030 | 2 | 162.000 | 13.353 | < 0.001 *

group:vision:support 0.189 | 0.095 | 2 | 162.000 | 1.228 | 0.295

*Posthoc tests: Vision*

**contrast | estimate | SE | df | t ratio | P-value**

EO - EC | -0.641 | 0.043 | 106.4 | -14.600 | < 0.001 *

EO - SRS | -0.610 | 0.043 | 75.0 | -14.001 | < 0.001 *

EC - SRS | 0.031 | 0.043 | 65.8 | 0.710 | 1.000

Degrees-of-freedom method: Satterthwaite

P value adjustment: Bonferroni method for 3 tests

*Posthoc tests: Group x Support*

**contrast | estimate | SE | df | t.ratio | P-value**

CNT firm - PTS firm | -0.463 | 0.085 | 54 | -5.436 | < 0.001 *

CNT SRP - PTS SRP | -0.886 | 0.111 | 54 | -7.940 | < 0.001 *

CNT firm - CNT SRP | -1.405 | 0.066 | 54 | -21.142 | < 0.001 *

PTS firm - PTS SRP | -1.828 | 0.105 | 54 | -17.398 | < 0.001 *

Degrees-of-freedom method: Satterthwaite

P value adjustment: Bonferroni method for 4 tests

*Posthoc tests: Vision X Support*

**contrast | estimate | SE | df | t.ratio | P-value**

EO firm - EC firm | -0.429 | 0.060 | 151 | -7.144 | < 0.001 *

EO firm - SRS firm | -0.486 | 0.059 | 168 | -8.130 | < 0.001 *

EC firm - SRS firm | -0.057 | 0.060 | 163 | -0.955 | 1.000

EO SRP - EC SRP | -0.852 | 0.060 | 151 | -14.175 | < 0.001 *

EO SRP - SRS SRP | -0.732 | 0.059 | 168 | -12.240 | < 0.001 *

EC SRP - SRS SRP | 0.119 | 0.060 | 163 | 1.990 | 0.434

EO firm - EO SRP | -1.393 | 0.078 | 121 | -17.823 | < 0.001 *

EC firm - EC SRP | -1.816 | 0.078 | 121 | -23.229 | < 0.001 *

SRS firm - SRS SRP | -1.639 | 0.078 | 121 | -20.969 | < 0.001 *

Degrees-of-freedom method: Satterthwaite

P value adjustment: bonferroni method for 9 tests

# Sensory Organisation Test: falls removed.

The analysis of the Sensory Organisation Test was repeated after removing the fall blocks (i.e. the blocks of trials in which participants had fallen in all three repetitions). The results of the primary analysis are largely confirmed.

**Type III Analysis of Variance Table with Satterthwaite's method**

**Sum Sq | Mean Sq | NumDF | DenDF | F value | P-value**

group 3.278 | 3.278 | 1 | 57.331 | 45.507 | < 0.001 *

vision 15.941 | 7.970 | 2 | 90.159 | 110.652 | < 0.001 *

support 43.377 | 43.377 | 1 | 56.698 | 602.199 | < 0.001 *

group:vision 0.198 | 0.099 | 2 | 90.159 | 1.374 | 0.258

group:support 0.141 | 0.141 | 1 | 56.698 | 1.957 | 0.167

vision:support 1.497 | 0.748 | 2 | 165.488 | 10.390 | < 0.001 *

group:vision:support 0.505 | 0.253 | 2 | 165.488 | 3.506 | 0.032 *

*Posthoc tests: Vision*

**contrast | estimate | SE | df | t.ratio | P-value**

EO - EC | -0.630 | 0.046 | 121.0 | -13.521 | < 0.001 *

EO - SRS | -0.552 | 0.049 | 94.4 | -11.257 | < 0.001 *

EC - SRS | 0.078 | 0.051 | 67.2 | 1.516 | 0.402

Degrees-of-freedom method: Satterthwaite

P value adjustment: Bonferroni method for 3 tests

*Posthoc tests: Vision X Support*

**contrast | estimate | SE | df | t.ratio | P-value**

EO firm - EC firm | -0.429 | 0.057 | 133 | -7.433 | < 0.001 *

EO firm - SRS firm | -0.486 | 0.058 | 145 | -8.321 | < 0.001 *

EC firm - SRS firm | -0.057 | 0.059 | 130 | -0.964 | 1.000

EO SRP - EC SRP | -0.832 | 0.070 | 162 | -11.774 | < 0.001 *

EO SRP - SRS SRP | -0.617 | 0.075 | 173 | -8.208 | < 0.001 *

EC SRP - SRS SRP | 0.214 | 0.080 | 149 | 2.669 | 0.076

EO firm - EO SRP | -1.312 | 0.074 | 119 | -17.647 | < 0.001 *

EC firm - EC SRP | -1.714 | 0.082 | 141 | -20.871 | < 0.001 *

SRS firm - SRS SRP | -1.443 | 0.085 | 157 | -16.824 | < 0.001 *

Degrees-of-freedom method: Satterthwaite

P value adjustment: Bonferroni method for 9 tests

*Posthoc tests: Group x Vision X Support*

**contrast | estimate | SE | df | t.ratio | P-value**

CNT firm EO - CNT firm EC | -0.483 | 0.061 | 133.1 | -7.824 | < 0.001 *

CNT firm EO - CNT firm SRS | -0.429 | 0.062 | 144.9 | -6.873 | < 0.001 *

CNT firm EC - CNT firm SRS | 0.053 | 0.063 | 130.2 | 0.842 | 1.000

PTS firm EO - PTS firm EC | -0.375 | 0.097 | 133.1 | -3.846 | 0.004 *

PTS firm EO - PTS firm SRS | -0.543 | 0.098 | 144.9 | -5.499 | < 0.001 *

PTS firm EC - PTS firm SRS | -0.168 | 0.100 | 130.2 | -1.674 | 1.000

CNT SRP EO - CNT SRP EC | -0.921 | 0.061 | 133.1 | -14.912 | < 0.001 *

CNT SRP EO - CNT SRP SRS | -0.794 | 0.062 | 144.9 | -12.698 | < 0.001 *

CNT SRP EC - CNT SRP SRS | 0.126 | 0.063 | 130.2 | 1.999 | 1.000

PTS SRP EO - PTS SRP EC | -0.743 | 0.127 | 166.3 | -5.846 | < 0.001 *

PTS SRP EO - PTS SRP SRS | -0.441 | 0.136 | 175.0 | -3.224 | 0.036 *

PTS SRP EC - PTS SRP SRS | 0.301 | 0.147 | 152.6 | 2.046 | 1.000

CNT firm EO - CNT SRP EO | -1.137 | 0.076 | 117.7 | -14.866 | < 0.001 *

CNT firm EC - CNT SRP EC | -1.575 | 0.076 | 117.7 | -20.587 | < 0.001 *

CNT firm SRS - CNT SRP SRS | -1.501 | 0.076 | 117.7 | -19.627 | < 0.001 *

PTS firm EO - PTS SRP EO | -1.486 | 0.127 | 119.5 | -11.659 | < 0.001 *

PTS firm EC - PTS SRP EC | -1.853 | 0.145 | 146.1 | -12.751 | < 0.001 *

PTS firm SRS - PTS SRP SRS | -1.384 | 0.153 | 163.1 | -9.015 | < 0.001 *

CNT firm EO - PTS firm EO | -0.460 | 0.110 | 75.8 | -4.184 | 0.001 *

CNT SRP EO - PTS SRP EO | -0.809 | 0.138 | 68.3 | -5.836 | < 0.001 *

CNT firm EC - PTS firm EC | -0.353 | 0.102 | 80.0 | -3.456 | 0.021 *

CNT SRP EC - PTS SRP EC | -0.631 | 0.139 | 85.1 | -4.529 | < 0.001 *

CNT firm SRS - PTS firm SRS| -0.574 | 0.113 | 69.8 | -5.063 | < 0.001 *

CNT SRP SRS - PTS SRP SRS | -0.457 | 0.155 | 79.8 | -2.948 | 0.100

Degrees-of-freedom method: Satterthwaite

P value adjustment: Bonferroni method for 24 tests

# Motor Control Test

**Type III Analysis of Variance Table with Satterthwaite's method**

**Sum Sq | Mean Sq | NumDF | DenDF | F value | P-value**

group 2.030 | 2.030 | 1 | 54.063 | 14.878 | < 0.001 *

dir 6.876 | 6.876 | 1 | 53.941 | 50.378 | < 0.001 *

amplitude 1.586 | 0.793 | 2 | 78.335 | 5.811 | 0.004 *

group:dir 0.716 | 0.716 | 1 | 53.941 | 5.246 | 0.025 *

group:ampl 0.133 | 0.066 | 2 | 78.335 | 0.489 | 0.614

dir:ampl 2.039 | 1.019 | 2 | 161.527 | 7.471 | < 0.001 *

group:dir:ampl 0.719 | 0.359 | 2 | 161.527 | 2.635 | 0.074

*Posthoc tests: Amplitude*

**contrast | estimate | SE | df | t.ratio | P-value**

small - medium | -0.201 | 0.072 | 63.1 | -2.770 | 0.022 *

small - large | -0.338 | 0.099 | 54.6 | -3.393 | 0.003 *

medium - large | -0.138 | 0.065 | 71.0 | -2.094 | 0.119

Degrees-of-freedom method: Satterthwaite

P value adjustment: Bonferroni method for 3 tests

*Posthoc tests: Group x Direction*

**contrast | estimate | SE | df | t.ratio | P-value**

CNT back - CNT forw | 0.419 | 0.093 | 53.9 | 4.499 | < 0.001 *

PTS back - PTS forw | 0.818 | 0.147 | 54.0 | 5.553 | < 0.001 *

CNT back - PTS back | 0.545 | 0.186 | 54.3 | 2.916 | 0.020 *

CNT forw - PTS forw | 0.944 | 0.234 | 54.0 | 4.034 | < 0.001 *

Degrees-of-freedom method: Satterthwaite

P value adjustment: Bonferroni method for 4 tests

*Posthoc tests: Direciton x Amplitude*

**contrast | estimate | SE | df | t.ratio | P-value**

back small - forw small | 0.374 | 0.108 | 118.9 | 3.440 | 0.007 *

back medium - forw medium | 0.701 | 0.107 | 114.3 | 6.527 | < 0.001 *

back large - forw large | 0.779 | 0.107 | 114.3 | 7.258 | < 0.001 *

back small - back medium | -0.364 | 0.091 | 121.0 | -3.972 | 0.001 *

back small - back large | -0.540 | 0.114 | 91.1 | -4.728 | < 0.001 *

back medium - back large | -0.176 | 0.085 | 130.2 | -2.069 | 0.364

forw small - forw medium | -0.037 | 0.090 | 119.1 | -0.413 | 1.000

forw small - forw large | -0.135 | 0.113 | 89.5 | -1.195 | 1.000

forw medium - forw large | -0.098 | 0.085 | 130.2 | -1.151 | 1.000

Degrees-of-freedom method: Satterthwaite

P value adjustment: Bonferroni method for 9 tests

# Visual dependence in DM1: falls removed.

The SOT showed that the COM sway was more significant in patients than controls even when standing with the eyes open, both on firm and moving platforms. So, there is a chance that differences between groups with closed eyes and the sway-referenced screen reflect the between-groups difference in quiet standing. Regression modelling was used to test this possibility.

The control analysis reported here repeats the primary analysis detailed in the main text after removing the trials in which patients had fallen.

The following linear regression models were tested:

COM_SRP_EC = COM_SRP_EO + group (1)

with COM_SRP_EC: natural logarithm (ln) of the normalised amplitude of the COM sway on the sway-referenced platform (SRP) with the eyes closed (EC)

COM_SRP_EC: ln m of the normalised amplitude of the COM sway on the SRP with the eyes open (EO)

group: control (0), patient (1)

and COM_SRP_SRS = COM_SRP_EO + group (2)

with COM_SRP_SRS: ln of the normalised amplitude of the COM sway on the SRP, with the sway-referenced screen (SRS)

Eight and nine trials were removed in models 1 and 2, respectively.

Models’ results:

**COM_SRP_EC = COM_SRP_EO + group**

Coefficients:

**Estimate | Std. Error | t value | P-value**

(Intercept) 2.712 | 0.243 | 11.124 | < 0.001 *

**COM_SRP_EO** 0.289 | 0.094 | 3.047 | 0.003 *

**PTS** 0.353 | 0.127 | 2.779 | 0.007 *

Residual standard error: 0.302 on 45 degrees of freedom

Multiple R-squared: 0.383, Adjusted R-squared: 0.356

F-statistic: 14 on 2 and 45 DF, p-value: < 0.001

**COM_SRP_SRS = COM_SRP_EO + group**

Coefficients:

**Estimate | Std. Error | t value | P-value**

(Intercept) 2.437 | 0.317 | 7.677 | < 0.001 *

**COM_SRP_EO** 0.348 | 0.123 | 2.816 | 0.007

**PTS** 0.139 | 0.164 | 0.848 | 0.401

---

Signif. codes: 0 ‘***’ 0.001 ‘**’ 0.01 ‘*’ 0.05 ‘.’ 0.1 ‘ ’ 1

Residual standard error: 0.380 on 44 degrees of freedom

Multiple R-squared: 0.203, Adjusted R-squared: 0.167

F-statistic: 5.628 on 2 and 44 DF, p-value: 0.006
